# A Chemical Facilitator of Plant–Fungus Communication

**DOI:** 10.1371/journal.pbio.0040241

**Published:** 2006-06-27

**Authors:** Liza Gross

Just beneath the surface of the arable floor, plants engage in an intimate exchange of life-sustaining elements with their subterranean fungal partners. In a mutually beneficial relationship fine-tuned over millions of years, soil fungi deliver essential nutrients to plants and receive photosynthetically produced carbon compounds in return. Arbuscular mycorrhizal fungi extend the plant's root system via long microscopic filaments (called hyphae) that zigzag through roots and soil in an elaborate underground network of mycelium. How the fungus knows a plant is near and that it should produce hyphal branches, however, is only beginning to be worked out.

Plant roots release thousands of compounds—from amino acids, hormones, and sugars to inorganic acids, inorganic ions, and carbon dioxide—complicating efforts to figure out which ones trigger hyphal branching. Previous studies found branching factors in carrots that stimulate the cell division required for branching. Just last year, researchers in Japan reported that the branching factor of a legume *(Lotus japonicus)* contains a strigolactone that stimulates hyphal branching in the *Gigaspora margarita* fungus. Strigolactones are a class of compounds that stimulate the germination of parasitic plants, which also must recognize their plant host to survive.

In a new study, Arnaud Besserer, Nathalie Séjalon-Delmas, and colleagues report that strigolactones may play a much wider role as branching stimulants, facilitating this critical step toward symbiosis for a broad range of plant–fungus partners. They also show that the compound increases the activity of mitochondria, the cell's power generators, just before branching begins.

The researchers first confirmed that carrot branching factors induce germination of the parasitic *Orobanche* weed. *Orobanche* seeds treated with the carrot branching factor and a synthetic analog of strigolactone (GR24) germinated at far higher rates than controls. Since other compounds can also stimulate *Orobanche* germination, the researchers tested their ability to stimulate hyphal branching. But only GR24 had any effect on the fungus *Gigaspora rosea*.

With confirmation that strigolactones can induce hyphal branching, Besserer et al. wondered how widespread this capability might be. In the 2005 study, the strigolactone-containing branching factor was found in *L. japonicus*, a dicot (seedlings emerge from their embryonic shell with two leaves). To see if more-distant plants also display strigolactone activity, Besserer et al. studied the monocot *Sorghum* (which emerges with one leaf). They grew *Sorghum* seedlings, collected root exudates after a month, then applied extracts to germinated *G. rosea* spores. Extracts that stimulated the most hyphal branching contained sorgolactone—one of two strigolactones associated with *Sorghum*—indicating a possible general role for the chemical signal.

Previous studies found that prior to *G. rosea* branching after treatment with carrot branching factors, fungal cells produced more mitochondria and increased their respiration rate. To see whether branching factor strigolactones stimulate respiration (which provides the energy needed for growth by breaking down lipids), Besserer et al. marked hyphae with fluorescent markers and treated them with GR24. Fluorescent microscopy revealed that the density of mitochondria—where respiration takes place—had indeed increased by 23% just an hour after G24 treatment. The shape and location of mitochondria had also changed significantly: the normally spherical, randomly distributed organelles flattened out along the axis of the hyphae.

Finally, the researchers showed that strigolactones exerted similar effects on evolutionarily distant fungi. G24 and another synthetic strigolactone (GR7) both increased the respiration rate of the fungus *Glomus intraradices*, resulting from a 30% increase in mitochondria—which had assumed the threadlike configuration observed in G. rosea.

How plants synthesize strigolactones is unclear, but the chemical's role in mediating essential soil–root interactions—not just for parasitic plants but also for symbiotic fungi and plants—suggests it may be found throughout the plant kingdom. Plant biologists can now test the hypothesis that strigolactones are a prerequisite for mycorrhizal symbiosis by studying mutant strigolactone-deficient plants. Understanding how this class of compounds establishes the mycelial network that sustains fungus-dependent plants is hardly an academic enterprise: some 80% of plant species, many living in endangered forest ecosystems, engage in these symbiotic partnerships. To learn more about strigolactone signaling, see the related Primer at DOI: 10.1371/journal.pbio.0040239.

## 

**Figure:**
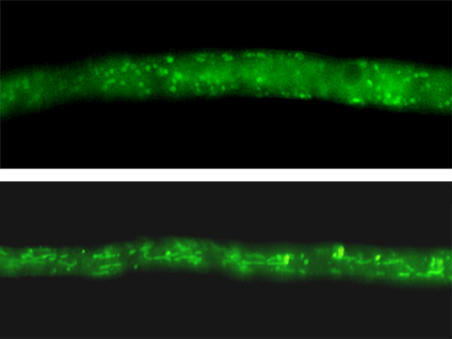
Chemicals from plant roots change the shape (from spherical, top, to threadlike, bottom) and increase the density of mitochondria in fungus, setting the stage for a symbiotic partnership.

